# The Missing lnc(RNA) between the pancreatic β-cell and diabetes

**DOI:** 10.3389/fgene.2014.00200

**Published:** 2014-07-01

**Authors:** Vasumathi Kameswaran, Klaus H. Kaestner

**Affiliations:** Department of Genetics and Institute for Diabetes, Obesity and Metabolism, Perelman School of Medicine, University of PennsylvaniaPhiladelphia, PA, USA

**Keywords:** lncRNA, β-cell biology, diabetes mellitus, imprinting control region (ICR), *MEG3*

## Abstract

Diabetes mellitus represents a group of complex metabolic diseases that result in impaired glucose homeostasis, which includes destruction of β-cells or the failure of these insulin-secreting cells to compensate for increased metabolic demand. Despite a strong interest in characterizing the transcriptome of the different human islet cell types to understand the molecular basis of diabetes, very little attention has been paid to the role of long non-coding RNAs (lncRNAs) and their contribution to this disease. Here we summarize the growing evidence for the potential role of these lncRNAs in β-cell function and dysregulation in diabetes, with a focus on imprinted genomic loci.

## INTRODUCTION

Recent technological advances in the field of genome sequencing have paved the way for a new appreciation of non-coding RNAs in gene regulation. Ultra high-throughput transcriptome analyses have revealed that the vast majority of the genome is transcribed, with two-thirds of the human genome covered by processed transcripts, of which only a small fraction (<2%) is translated into proteins (Djebali et al., 2012). The identification of several common genomic and functional features of these untranslated RNAs has led to their categorization into various classes of non-coding RNAs. One such class that has been the focus of extensive research is that of long non-coding RNAs (lncRNAs).

LncRNAs are defined as transcripts longer than 200 bp that lack protein-coding potential (Guttman et al., 2009; Derrien et al., 2012; Batista and Chang, 2013; Fatica and Bozzoni, 2014). Like messenger RNAs, lncRNAs typically have multiple exons, are processed using canonical splice sites, and may exist as several isoforms (Ponjavic et al., 2007; Cabili et al., 2011; Derrien et al., 2012). In contrast to mRNAs, lncRNAs preferentially display nuclear localization, consistent with their proposed function in chromatin organization and regulation of gene expression (Khalil et al., 2009; Zhao et al., 2010; Derrien et al., 2012; Guttman and Rinn, 2012; Rinn and Chang, 2012; Fatica and Bozzoni, 2014).

Similar to protein-coding genes, lncRNA-encoding genes are marked by chromatin signatures typical of active transcription in the cell types where they are expressed, consisting of H3K4me3 (trimethylated lysine 4 in histone H3) at the promoter, followed by H3K36me3 along the transcribed regions (so-called “K4–K36 domains”; Guttman et al., 2009; Khalil et al., 2009; Cabili et al., 2011; Guttman and Rinn, 2012; Rinn and Chang, 2012). While lncRNA exons display weaker evolutionary conservation than those of protein-coding genes, there is evidence of positive selection for a subset of lncRNAs, which may be driven by constraints to maintain secondary structure required for functional interactions with their targets (Ponjavic et al., 2007; Guttman et al., 2009; Cabili et al., 2011; Ulitsky et al., 2011; Derrien et al., 2012). In contrast, the promoters of lncRNAs are as highly conserved as those of protein-coding genes (Carninci et al., 2005; Ponjavic et al., 2007; Guttman et al., 2009; Derrien et al., 2012; Batista and Chang, 2013). Despite their overall lower expression levels, lncRNAs exhibit a higher degree of tissue specificity compared to average protein-coding genes (Mercer et al., 2008; Cabili et al., 2011; Derrien et al., 2012; Batista and Chang, 2013; Fatica and Bozzoni, 2014).

Through numerous studies, several general principles of lncRNA function have emerged. LncRNAs have been shown to function both in *cis*, i.e., locally close to the site of their production, and in *trans*, i.e., at sites on other chromosomes. LncRNAs have been proposed to act as scaffolds for chromatin modifiers, blockers of transcription, antisense RNAs, microRNA sponges, protein decoys, and enhancers (Cech and Steitz, 2014; Fatica and Bozzoni, 2014). In fact, the act of transcription of a lncRNA itself can interfere with the regulatory function of a regulatory DNA sequence, as exemplified in yeast (Martens et al., 2004) and in mammalian imprinting (Latos et al., 2012). As a result of their diverse functions in multiple tissues, mis-regulation of lncRNAs can lead to failure of normal development and, consequently, to disease. Mammalian chromatin modifiers such as the repressive *polycomb* complexes often lack their own specific DNA-binding domains but instead contain RNA-binding elements. LncRNAs can play critical roles in directing these repressive chromatin modifying complexes to their target regions (Bernstein and Allis, 2005; Rinn et al., 2007; Zhao et al., 2010). One such example is the Foxf1-adjacent, non-coding developmental regulatory RNA (*Fendrr*), a lncRNA that interacts with the polycomb repressive complex 2 (PRC2) and is critical for heart development and function (Grote et al., 2013). Similarly, the well-characterized *HOTAIR* lncRNA, which is transcribed from the *HOXC* locus, is highly upregulated in primary breast tumors and was shown to function through the silencing of tumor suppressor genes in a PRC2-dependent manner [Gupta et al., 2010; See Maass et al. (2014) for a list of lncRNAs currently implicated in human diseases]. Taken together, these features suggest that lncRNAs and other non-coding RNA species may play an essential role in defining organismal complexity (Mattick and Makunin, 2006; Taft et al., 2007).

These findings raise the possibility that lncRNAs and other non-coding RNAs may be exciting molecular candidates to account for the unresolved genetic risk in complex diseases such as diabetes (Medici et al., 1999; Hyttinen et al., 2003). Diabetes mellitus represents a group of metabolic diseases that result in impaired glucose homeostasis. In the case of type 1 diabetes (T1D), metabolic impairment is the result of autoimmune destruction of insulin-producing pancreatic β-cells. In type 2 diabetes (T2D), the most prevalent form of the disease, the defect in glucose metabolism is the result of decreased sensitivity of peripheral tissues to insulin action, accompanied by failure of β-cells to compensate for the increased metabolic demand (Zimmet et al., 2001). Together, these diseases affect over 25 million Americans and account for $176 billion in healthcare costs per year in the US alone (Association, 2013), necessitating the pursuit of more effective and personalized treatments.

Significant efforts have been made to attain a better understanding of the causes of diabetes at the molecular level. Linkage analysis of affected families led to the successful identification of causal gene mutation in several rare, Mendelian forms of the disease (Fajans et al., 2001; O’Rahilly, 2009). However, large-scale efforts to identify DNA variants associated with more common forms of diabetes through genome-wide association studies (GWAS) have predominantly identified candidate variants that lie in non-coding regions and with as yet unknown functions (McCarthy, 2010). Thus, to improve our current understanding of the molecular basis of diabetes mellitus and to develop better treatment strategies, we need to carefully characterize the transcriptome of pancreatic β-cells, with a focus on elucidating the functions of non-coding transcripts. In this review, we present a summary of recent evidence for a role of lncRNAs in the regulation of β-cell function and their potential contribution to the pathogenesis of diabetes.

## β-CELL lncRNAs

The most comprehensive catalog of human lncRNAs expressed in β-cells published thus far is that by Morán et al. (2012). In this study, the authors profiled whole islet and FACS-sorted β-cells and identified 1,128 distinct transcripts that displayed many of the typical properties of lncRNAs described above, including the “K4–K36” histone modification domains, lack of protein-coding potential, and non-uniform expression levels among tissues. Most notably, the lncRNAs identified were roughly five times more islet-specific compared to general protein-coding genes, and the vast majority had orthologous genes in the mouse genome. Ku et al. (2012) similarly characterized mouse islet- and β-cell-specific transcripts and identified 1,359 high-confidence lncRNAs with several of the aforementioned properties. Using high-throughput transcriptome analysis of sorted human islets, lncRNAs expressed in α-cells have also been identified (Bramswig et al., 2013).

Of particular interest was the fact that lncRNAs were often found in proximity to critical islet-specific transcription factors (Ku et al., 2012; Morán et al., 2012). Thus, protein-coding genes adjacent to islet-enriched lncRNAs were also more likely to be islet-specific than the average protein-coding gene (Morán et al., 2012). This correlation has led to the suggestion that lncRNAs and nearby protein-coding genes share common regulatory elements. Indeed, lncRNAs were often found in large regions of open chromatin that were uniquely associated with protein-coding genes expressed highly in islets (Gaulton et al., 2010).

The temporal expression of islet lncRNAs has also been studied by Morán et al. (2012) in human embryonic pancreases as well as in a stepwise *in vitro* β-cell differentiation model using human embryonic stem (ES) cells (developed by Kroon et al., 2008). Unlike some lncRNAs that are known to be critical to early stages of embryonic development (Guttman et al., 2011; Grote et al., 2013), the expression of a majority of islet lncRNAs identified in this study (Morán et al., 2012) is restricted to differentiated, mature endocrine cells. The orthologous mouse lncRNAs (e.g., *Mi-Lnc80*) exhibit similar cell- and stage-specific expression.

The characteristics of these islet lncRNAs imply a role for these RNAs in mature β-cell function. To test this hypothesis, Morán et al. (2012) used short hairpin RNAs (shRNAs) to suppress the activity of one such lncRNA transcript in the human EndoC-βH1 β-cell line (Ravassard et al., 2011). From a panel of known islet-specific transcripts, the authors identified *GLIS3* as a downstream target of *HI-LNC25*, a lncRNA that shares a regulatory domain with *MAFB*. Variants at the *GLIS3* locus are associated with different risks for T1D (Barrett et al., 2009), elevated fasting glucose levels (Dupuis et al., 2010), as well as T2D (Cho et al., 2012). Loss-of function studies suggest that *GLIS3* encodes a transcription factor critical for regulating the expression of insulin and several key islet-transcription factors, and may confer risk for both T1D and T2D by resulting in diminished β-cell numbers and by promoting the formation of a pro-apoptotic splice variant of the protein *Bim* (Kang et al., 2009; Nogueira et al., 2013; ZeRuth et al., 2013). However, the shRNA-mediated decrease in *GLIS3* mRNA levels had no impact on glucose-stimulated insulin secretion or insulin transcript levels in the transduced EndoC-βH1 β-cell line, possibly because this cell line does not recapitulate all aspects of β-cell function *in vivo*. Additionally, only a minor fraction of β-cell expressed lncRNAs was responsive to elevated glucose levels in human islets.

As previously noted, several risk variants for common forms of diabetes identified by GWAS do not change the protein-coding potential of known genes, suggesting that they might affect as yet unidentified regulatory elements (McCarthy, 2010). Using a computational tool known as MAGENTA to search for enrichment of genetic associations in a predefined set of genes (Segrè et al., 2010), Morán et al. (2012) determined that the islet lncRNA genes identified in their study were in fact highly enriched for risk alleles associated with T2D and related phenotypes, further underscoring the need to interrogate the function of these RNAs in β-cell biology.

Overall, these studies highlighted lncRNAs as a major component of the β-cell transcriptome that is cell-type-specific, developmentally regulated, and evolutionarily conserved with strong associations to disease risk. However, it still remains to be determined how these lncRNAs may contribute to β-cell function, and if their mis-regulation may play a role in diabetes. Their expression in EndoC-βH1 cells and mouse islets provides additional platforms to evaluate their function in a systematic and comprehensive manner. Future studies will also need to address the question of whether the lncRNAs identified thus far act in *cis* (on neighboring islet protein-coding genes) or in *trans* to exert their function.

## IMPRINTING

Some of the best characterized lncRNAs to date were first uncovered in early studies of imprinting and dosage compensation of the X-chromosome (Brannan et al., 1990; Brown et al., 1991; Fatica and Bozzoni, 2014). Imprinting refers to the biased expression of genes depending on the parental origin of the chromosome. This process is tightly regulated, typically through epigenetic modifications such as DNA methylation at *cis*-acting elements known as “imprinting control regions” (ICRs), to establish and maintain mono-allelic expression of specific genes (Thorvaldsen and Bartolomei, 2007). Methylation at the ICRs is maintained despite active demethylation and dynamic reprogramming in the newly formed zygote, and is only altered during establishment of methylation pattern in a sex-specific manner during primordial germ cell development (Bartolomei and Ferguson-Smith, 2011). Imprinted loci are generally found in large clusters, where both maternally- and paternally expressed genes are interspersed. Frequently, the protein-coding genes are expressed from one parental allele, while non-coding genes are expressed from the other (Barlow, 2011). LncRNAs play an essential role in the regulation of mono-allelic expression, either by acting in *cis* as an antisense molecule to block the transcriptional machinery, or by directly recruiting repressive chromatin modifiers to silence reciprocally expressed genes (Lee and Bartolomei, 2013).

While imprinting is most extensively studied in the context of fetal development, tissue-specific regulation in adult tissues has also been observed (Barlow, 2011; Lee and Bartolomei, 2013). As a result, several imprinted genes are also implicated in human diseases that arise from somatic tissues. One such example is that of the maternally expressed adipose tissue transcription factor, *KLF14* (Parker-Katiraee et al., 2007), which is associated with risk for both T2D and high-density lipoprotein disorders (Teslovich et al., 2010; Voight et al., 2010; Small et al., 2011). Perhaps the functionally haploid nature of these loci results in their increased likelihood to be associated with susceptibility to disease, as mutations in these genes, when found on the maternal chromosome that is expressed, cannot be “covered” by the gene from the other, silenced paternal allele. This may be particularly true for metabolic disorders, as several imprinted genes encode dosage-sensitive proteins related to growth factors and energy metabolism. Interestingly, several risk variants for type 1 and type 2 diabetes identified through GWAS are located in imprinted loci including *KCNQ1*, *MEG3, PLAGL1*, and *GRB10*. A few of these are discussed below in the context of islet and β-cell function.

### *DLK1–MEG3* LOCUS

Recently, we identified the maternally expressed non-coding RNAs of the imprinted *DLK1*–*MEG3* locus as down-regulated in human islets from T2D donors (Kameswaran et al., 2014). This gene cluster is located on human 14q32 (mouse chromosome 12) and contains three paternally expressed protein-coding genes, *DLK1, RTL1*, and* DIO3*. *DLK1* is a non-canonical Notch ligand that is expressed in many embryonic tissues (Falix et al., 2012) and is a well-established negative regulator of adipocyte differentiation (Smas and Sul, 1993; Mitterberger et al., 2012; Abdallah et al., 2013). *DLK1* is highly expressed in human and mouse β-cells (Tornehave et al., 1996; Dorrell et al., 2011; Appelbe et al., 2013). While *DLK1* was demonstrated to be stimulated by growth hormone and prolactin expression in rat islets, including during pregnancy, it is not directly responsible for the mitogenic effects of these hormones on islets (Carlsson et al., 1997; Friedrichsen et al., 2003). Additionally, loss of expression of *Dlk1* in unchallenged mouse β-cells does not cause any observable phenotype (Appelbe et al., 2013). *Rtl1* (*Retrotransposon-like 1*) is critical for normal placental development and its loss results in severe developmental defects and late-fetal lethality (Sekita et al., 2008).

The maternally expressed genes are all non-coding RNAs, consisting of the lncRNA, *Maternally Expressed Gene 3* (*MEG3*, known as *Gtl2* in mice), as well as a large cluster of microRNAs (miRNAs) and snoRNAs (Schmidt et al., 2000; Seitz et al., 2004; da Rocha et al., 2008). In several tissues, including human islets, the non-coding RNAs are all derived from a single transcript that initiates from the *MEG3* promoter (Tierling et al., 2006; da Rocha et al., 2008; Kameswaran et al., 2014).

**FIGURE 1:**
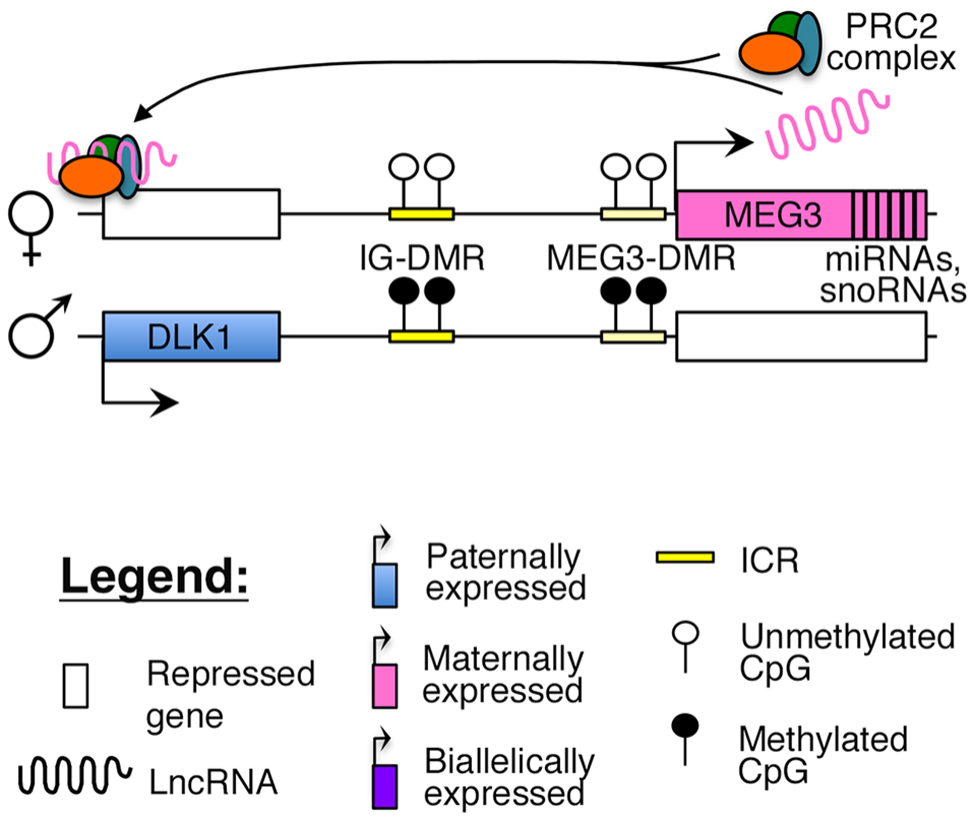
**Proposed model of imprinting at the *DLK1–MEG3* locus:** the *DLK1–MEG3* imprinted region contains a primary ICR (IG-DMR) and secondary (*MEG3–*DMR) ICR that overlaps with the promoter of the *MEG3*. Both ICRs are paternally methylated. In mouse ES cells, the *Meg3* lncRNA is believed to direct PRC2 mediated silencing of *Dlk1* (Zhao et al., 2010).

Reciprocal imprinting is established by methylation of two differentially methylated regions (DMRs) on the paternal allele, one located ∼13 kb upstream of the *MEG3* transcription start site (IG-DMR), and the other overlapping with the promoter of the *MEG3* poly cistronic transcript (*MEG3*-DMR; **Figure 1**). While the IG-DMR is the primary ICR for this imprinted cluster, the *MEG3*-DMR is also critical to regulating and maintaining imprinting at this region (Kagami et al., 2010). Failure to maintain imprinting at this locus can lead to either maternal or paternal uniparental disomy (UPD) of chromosome 14, which causes distinct and severe developmental disorders (Kagami et al., 2008).

Increased methylation of the *MEG3*-DMR and related loss of *MEG3* expression has been observed in several human cancers, such as pituitary and renal cell cancers and multiple myeloma (Zhao et al., 2005; Kawakami et al., 2006; Benetatos et al., 2008) to name a few (further reviewed by Benetatos et al., 2011). These studies, coupled with *in vitro* experiments, suggest that *MEG3* functions as a tumor suppressor by activating p53, in a manner dependent upon the secondary structure of the *MEG3* RNA (Zhou et al., 2007, 2012). Furthermore, decreased expression of *MEG3* and hypermethylation of the DMRs may single-handedly explain the subtle phenotypic differences between induced pluripotent stem cells (iPSCs) and ES cells, such as the decreased efficiency in generating chimeric mice from iPSCs (Stadtfeld et al., 2010).

Similar to the aforementioned examples, decreased expression of *MEG3* and the associated miRNAs in T2D islets strongly correlates with hypermethylation of the *MEG3*-DMR (Kameswaran et al., 2014). Additionally, a single nucleotide polymorphism (SNP) (rs941576) located in an intron of *MEG3* was found to be associated with T1D, with the risk allele being transmitted more frequently from the father than the mother of the affected offspring (Wallace et al., 2010). Overall, these examples provide compelling evidence for the importance of *MEG3* and the regulation of this imprinted region in several diseases. Despite the strong disease association of this lncRNA, and the fact that genes in this imprinted cluster are very highly expressed in human β-cells (Dorrell et al., 2011; Kameswaran et al., 2014), there are currently no postulated mechanisms for its potential role in β-cell function and diabetes pathogenesis.

Recent studies have suggested that similar to other nuclear lncRNAs, *MEG3* also directly interacts with the PRC2 complex in ES cells to guide the repressive histone modification mark H3K27me3 to its target sites (Zhao et al., 2010; Kaneko et al., 2014). One study identified *Dlk1* as a direct target of the *Meg3*-PRC2 complex in mouse ES cells (**Figure 1**), although this finding could not be replicated in *MEG3*-expressing human iPSCs, where *MEG3* was found to function in *trans* (Zhao et al., 2010; Kaneko et al., 2014). A careful characterization of *MEG3-*PRC2 complex targets in adult pancreatic islets will provide better insights into the role of this lncRNA in β-cell function.

### *KCNQ1* LOCUS

The *KCNQ1* gene, encoding a voltage-gated potassium channel, has been of great interest to the β-cell biology field due to its strong disease association. The gene is located in an imprinted locus on human 11p15.5, adjacent to another independently regulated imprinted locus, *H19–IGF2*. This region was implicated as a molecular candidate for Beckwith–Wiedemann syndrome (BWS), a disorder characterized by prenatal macrosomia, predisposition for tumor development and frequently, hyperinsulinemic hypoglycemia (Lee et al., 1997, 1999; Hussain et al., 2005). This imprinted region consists of several conserved, maternally expressed protein-coding genes, such as the cell cycle inhibitor *CDKN1C*, and a paternally expressed antisense lncRNA, *KCNQ1* overlapping transcript1 (*KCNQ1OT1*; Monk et al., 2006). Loss of imprinting in this locus can lead to the suppression of *CDKN1C*, which is sufficient to cause re-entry of adult human β-cells into the cell cycle (Avrahami et al., 2014).

**FIGURE 2:**
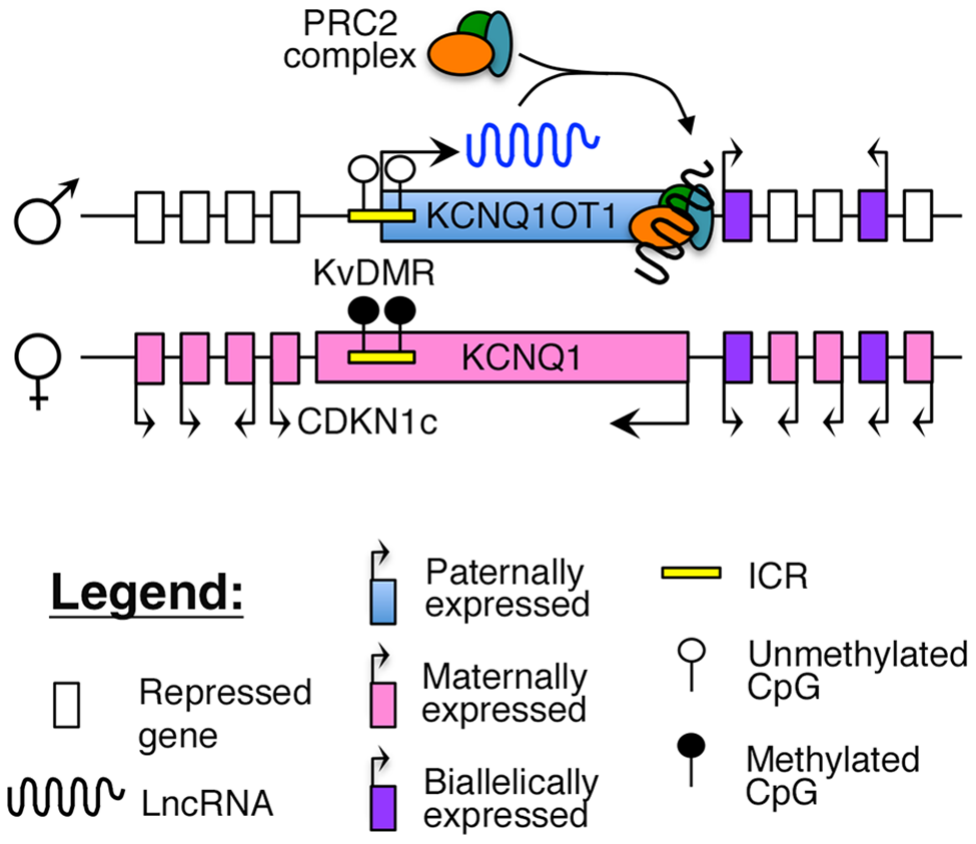
**Proposed model of imprinting at the *KCNQ1* locus:** the *KCNQ1OT1* lncRNA is expressed from the paternally unmethylated KvDMR ICR, which is methylated on the maternal allele. Recent evidence suggests that *KCNQ1OT1* can directly recruit the PRC2 complex and facilitate intra-chromosomal looping to the *KCNQ1* promoter (Zhang et al., 2014).

Imprinting of this region is maintained by a maternally methylated ICR, known as the KvDMR, which is also the promoter for *KCNQ1OT1* (**Figure 2**). To maintain appropriate mono-allelic expression of imprinted genes in this locus, the KvDMR is hypomethylated on the paternal allele, leading to expression of the *KCNQ1OT1* lncRNA and subsequent repression of the maternal, protein-coding genes on the same allele (Fitzpatrick et al., 2002; Ideraabdullah et al., 2008), possibly by facilitating intra-chromasomal looping to direct the repressive PRC2 complex to their promoter (**Figure 2**; Zhao et al., 2010; Zhang et al., 2014).

The *KCNQ1* locus harbors at least two independently identified and replicated GWAS signals at SNPs located in the intron of the *KCNQ1* gene (rs2237892), with one overlapping the *KCNQ1OT1* lncRNA (rs231362; Unoki et al., 2008; Yasuda et al., 2008; Kong et al., 2009; Voight et al., 2010). Additional SNPs in this gene, such as rs2237895, are also reported to be associated with T2D risk in specific ethnic populations (Unoki et al., 2008). While these SNPs are predicted to confer risk for diabetes only when maternally inherited (Kong et al., 2009), the risk alleles do not correlate with each other (Kong et al., 2009; Voight et al., 2010) and have opposing effects on docking of insulin granules (Rosengren et al., 2012).

To investigate how these T2D risk variants may affect allelic expression and imprinting of this region, Travers et al. (2013) correlated the risk SNP genotypes with DNA methylation and expression patterns of the imprinted genes in human fetal pancreas and adult islets. This study revealed that fetal samples homozygous for the rs2237895 risk allele had marginally increased methylation levels at the KvDMR region. As this was not observed in the adult, these results suggest that effects of the risk allele are likely be established during early stages of islet development, as *KCNQ1* and *KCNQ1OT1* are only imprinted in fetal but not adult tissues (Monk et al., 2006; Travers et al., 2013). Overall, this study proposes a model whereby each risk allele for the rs2237895 SNP leads to increased methylation of the KvDMR, and consequently, decreased expression of *KCNQ1OT1*. However, there was no observable difference in *KCNQ1* or *KCNQ1OT1* expression in samples used for this study. On the contrary, *KCNQ1OT1* transcript levels have been shown to be significantly elevated in T2D islets (where SNP genotype was not determined; Morán et al., 2012), which parallels an overall decrease in methylation at several tested CpGs near the *KCNQ1* gene (Dayeh et al., 2014). Thus, the interpretation of variants to disease pathology at this region has been contradictory and challenging. Nevertheless, the regulation of this locus and the lncRNA *KCNQ1OT1* remains relevant to β-cell biology and T2D pathogenesis.

### *H19–IGF2* LOCUS

**FIGURE 3:**
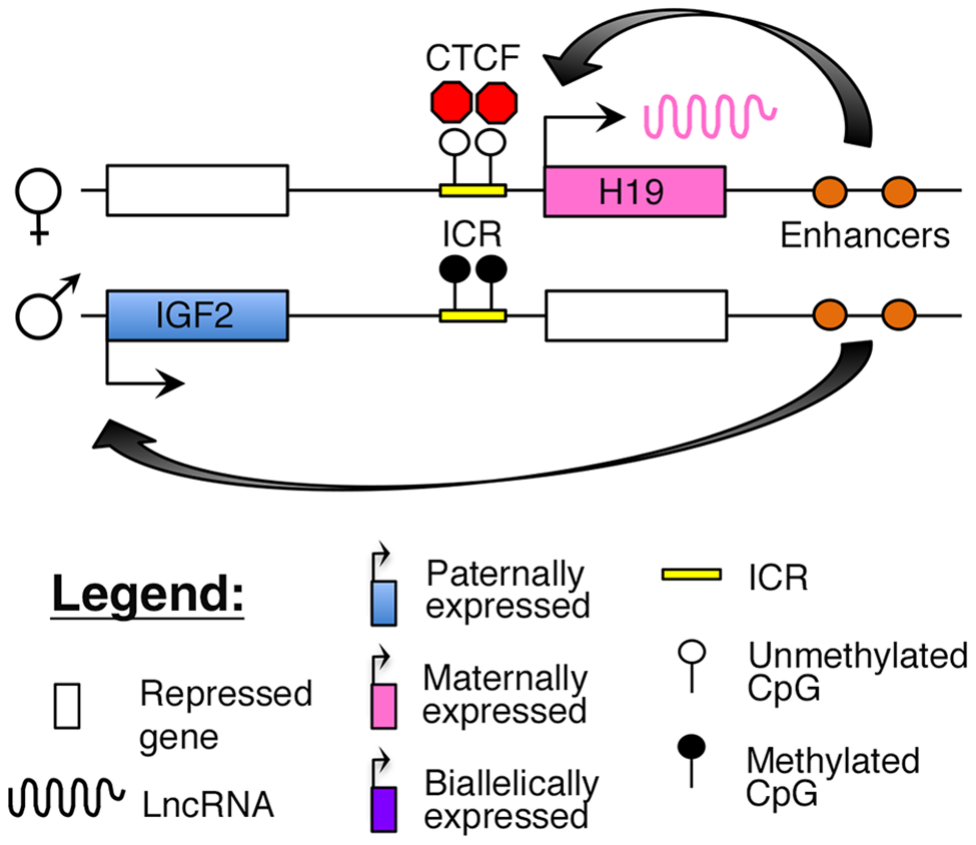
**Proposed model of imprinting at the *H19–IGF2* locus:** the *H19–IGF2* locus consists of a paternally methylated ICR. On the maternal allele, this ICR is unmethylated and is bound by the insulator protein CTCF that prevents access of the *IGF2* promoter to downstream enhancers.

The *H19–IGF2* locus resides adjacent to the *KCNQ1* region on human 11p15.5. The region consists of the paternally expressed *insulin-like growth factor 2* (*IGF2*) gene and maternally expressed *H19* lncRNA (Brannan et al., 1990; DeChiara et al., 1990; Bartolomei et al., 1991). The *IGF2* protein functions as a growth factor essential for embryonic development (DeChiara et al., 1990), whereas *H19* may function as a tumor suppressor (Hao et al., 1993). Imprinting at this locus is maintained by an ICR, which is selectively methylated on the paternal allele. The insulator protein, CCCTC-binding factor (*CTCF*), binds to critical regulatory regions in the unmethylated ICR on the maternal allele, thus blocking access of downstream enhancers to the *IGF2* promoter (**Figure 3**; Stadnick et al., 1999; Bell and Felsenfeld, 2000; Engel et al., 2004).

Loss of methylation at the *H19/IGF2* ICR results in short body length and low birth weight, both in rodent models (DeChiara et al., 1990) as well as in humans, such as patients with Silver-Russell syndrome, a developmental disorder characterized by intrauterine and postnatal growth retardation (Gicquel et al., 2005). This has also been observed in humans who were periconceptually exposed to famine (Heijmans et al., 2008). There is growing evidence that intra-uterine exposure to malnutrition can predispose the offspring to metabolic complications including β-cell dysfunction and diabetes later in life (Ravelli et al., 1998; Roseboom et al., 2006). This theory is commonly referred to as the “thrifty phenotype hypothesis” (Hales, 2001) and is thought to be mediated primarily through environmentally induced epigenetic changes to key metabolic regulators (Park et al., 2008; Bramswig and Kaestner, 2012). However, first and second generation progeny of mice exposed to gestational diabetes were found to have impaired glucose tolerance with hypermethylation of the *H19* ICR in islets (Ding et al., 2012). These contradicting observations may be a result of different nutrient availability that the developing fetus was exposed to, as well as the varying lengths of exposure. The above studies suggest that the *H19–IGF2* locus is highly responsive to these changes in the intrauterine milieu and may represent a prognostic marker of metabolic complications later in life.

Hypermethylation of the *H19–IGF2* ICR has been observed in some cases of BWS (Ohlsson et al., 1993), as well as in focal congenital hyperinsulinism (FoCHI), a glucose metabolism disorder characterized by unbridled insulin secretion from hyperplastic islet cells and consequent hypoglycemia (de Lonlay et al., 1997). Increased methylation at this ICR would be predicted to result in decreased *H19* expression, loss of imprinting at this region and a concomitant increase in *IGF2* expression. Although over-expression of *IGF2* in mouse β-cells recapitulates the FoCHI phenotype (Devedjian et al., 2000), *IGF2* expression was variable in human FoCHI lesions (Fournet et al., 2001). On the contrary, *H19* transcript levels were consistently down-regulated in these cells, suggesting that *H19* may have an important regulatory role in restraining islet-proliferation. This hyperproliferative phenotype, accompanied by suppression of *H19* has also been reported in Wilms’ tumor (Cui et al., 1997). Taken together, the *H19* lncRNA may function as a critical regulator of β-cell function and proliferation either on its own or indirectly through the regulation of *IGF2* levels.

### *ZAC–HYMAI* LOCUS

**FIGURE 4:**
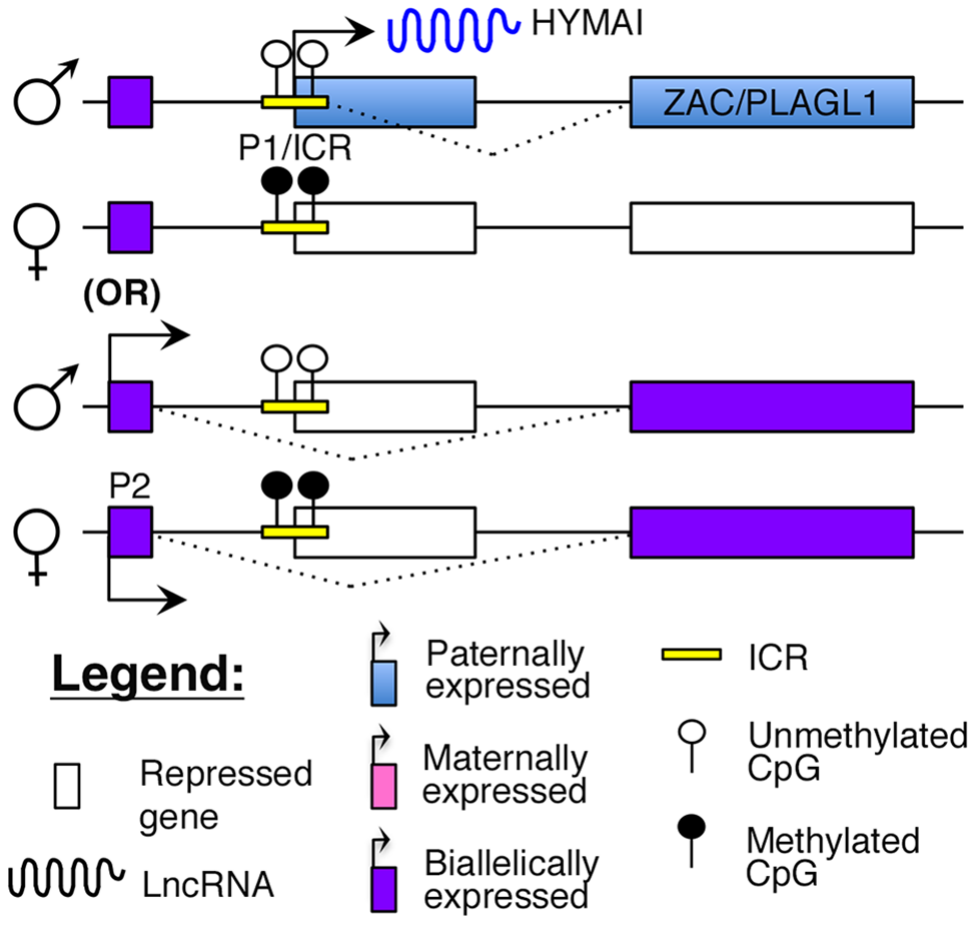
**Proposed model of imprinting at the *ZAC–HYMAI* locus:** *ZAC/PLAGL1* and the lncRNA *HYMAI* are both paternally expressed from a common promoter that is also the ICR. However, in some tissues, *ZAC* is biallelically expressed from an upstream promoter.

Transient neonatal diabetes (TNDM) is a rare form of diabetes mellitus characterized by hyperglycemia and low insulin levels within the first year of birth (Temple et al., 2000). This form of diabetes is distinct from T1D as there is no evidence for autoimmunity (Abramowicz et al., 1994; Shield et al., 1997). Although it usually resolves by 2 years of age, children with TNDM are at a higher risk of developing T2D later in life (Temple et al., 2000). The molecular cause of this disease was identified to be abnormal imprinting of chromosome 6q24, which encompasses the cell cycle regulator, *ZAC/PLAGL1*, and the lncRNA, *HYMAI* (Abramowicz et al., 1994; Arima et al., 2000; Gardner et al., 2000; Kamiya et al., 2000; Mackay et al., 2002). Both *ZAC* and *HYMAI* share a common imprinted promoter (P1 in **Figure 4**), which also serves as the ICR, and are expressed from the paternal allele (Arima et al., 2000; Mackay et al., 2002). However, tissue-specific usage of an alternative promoter (P2 in **Figure 4**) that drives biallelic expression of *ZAC* has also been reported (Valleley et al., 2007).

*ZAC* encodes a zinc finger protein that regulates apoptosis and cell cycle arrest (Spengler et al., 1997). The protein is expressed at very high levels in insulin-producing cells in the human fetal pancreas, but not adult islets (Du et al., 2011). *ZAC* can also function as a transcriptional activator of *CDKN1C* and *KCNQ1OT1* (Arima et al., 2005). *ZAC* is believed to control the induction of the pituitary adenylate cyclase-activating polypeptide (*PACAP*), a strong activator of glucose-stimulated insulin secretion (Yada et al., 1994; Ciani et al., 1999). These features of the *ZAC* gene make this a strong candidate for the pathogenesis of TNDM. However, the mechanism of imprinting and the function of *HYMAI* in the context of TNDM have yet to be established.

## *MALAT1*, AN ABUNDANT lncRNA

The *metastasis-associated lung adenocarcinoma transcript 1 (MALAT1)* is a highly conserved lncRNA that is mis-regulated in several tumors (Ji et al., 2003; Gutschner et al., 2013). *MALAT1* is very abundantly expressed (higher than many housekeeping genes) in multiple cell types, including the pancreas (Ji et al., 2003) and in purified human α- and β-cells (Dorrell et al., 2011). Additionally, *MALAT1* is encoded within an active enhancer cluster with several binding sites for islet-transcription factors (Pasquali et al., 2014), making this is an intriguing candidate for gene regulation in human islets.

*Metastasis-associated lung adenocarcinoma transcript 1* has several interacting partners through which it may mediate its function. One such interacting partner is DGCR8, a double-stranded RNA binding protein that together with Drosha mediates miRNA bioprocessing (Macias et al., 2012). *MALAT1* was found to be bound to Argonaute (Ago), the primary effector of miRNA function in HeLa cells (Weinmann et al., 2009). *MALAT1* was also found to be associated with Ago in human islets, suggesting that this lncRNA may be regulated by miRNAs in human cells (Kameswaran et al., 2014). In fact, we discovered several sequences that consisted of miRNAs fused to MALAT1 while assaying miRNAs and their targets that were bound to Ago in human islets. These chimeric reads were the result of ligation of two adjacent RNA species present in the RISC complex with Ago (Helwak et al., 2013), and proved that *MALAT1* is regulated by several miRNAs in human islets (Kameswaran et al., 2014).

*Metastasis-associated lung adenocarcinoma transcript 1* can also regulate gene expression through its association with different nuclear sub-compartments (Hutchinson et al., 2007; Yang et al., 2011; Gutschner et al., 2013). One example of this is *MALAT1* localization in nuclear speckles, which are nuclear domains where splicing factors are stored and post-transcriptionally modified (Hutchinson et al., 2007; Mao et al., 2011). Through the modification of critical splicing factors, *MALAT1* has been shown to contribute to alternative splicing (Tripathi et al., 2010). However, despite the abundance of this lncRNA and the early suggestions of its function from *in vitro* studies, mice lacking *MALAT1* displayed no obvious phenotype in the absence of additional pathological stressors and exhibit largely normal nuclear speckle formation and alternative splicing patterns (Eißmann et al., 2012; Nakagawa et al., 2012; Zhang et al., 2012). Thus, the role of this lncRNA remains to be determined.

## PERSPECTIVE

The exciting discovery of lncRNAs and the growing recognition of their involvement in human pathogenesis have added a new level of complexity to our understanding of gene regulation. However, due to the range of sequencing and bioinformatic tools currently available, the rate of discovery of new lncRNAs has surpassed our ability to examine their function. This gap between lncRNA gene discovery and function currently holds true in the field of β-cell biology as well, necessitating the systematic analysis of mouse and human islet lncRNAs identified to date (Ku et al., 2012; Morán et al., 2012).

Factors such as overlap between the human and mouse α- and β-cell lncRNA complements (Ku et al., 2012; Morán et al., 2012; Bramswig et al., 2013), degree of conservation, expression, associated protein-coding genes, and relative distance from GWAS SNP variants may be good early predictors of important lncRNAs. However, these parameters alone may underestimate other essential candidates, as some lncRNAs exhibit low primary sequence conservation despite crucial function (Nesterova et al., 2001), or, conversely, a dispensable function despite high sequence conservation and expression (Zhang et al., 2012). These observations emphasize the need for careful loss-of-function experiments in appropriate model systems induced by metabolic and/or inflammatory challenges to clearly understand the function of these lncRNAs. Although many of the human β-cell lncRNAs are expressed in the EndoC-βH1 cell line that somewhat resembles human β-cells *in vitro* (Ravassard et al., 2011), targeted deletion or inhibition in mouse and human islets may be necessary in some cases to reveal their function, as seen in the example of *HI-LNC25* discussed above (Morán et al., 2012).

While the loss-of-function of even abundant lncRNAs such as *MALAT1* may sometimes result in a lack of phenotype (Eißmann et al., 2012; Nakagawa et al., 2012; Zhang et al., 2012), lessons from the miRNA field suggest that additional physiological and environmental stressors may be necessary to truly elucidate the function of these non-coding RNAs (Mendell and Olson, 2012). Additionally, in order to study the role of lncRNAs in the context of loss-of-function, a careful analysis of the genomic location of the lncRNAs may be required to evaluate the best method of gene silencing, as targeted recombination may result in disruption of overlapping protein-coding transcripts or their regulatory domains, further confounding data interpretation.

Given the broad range of human diseases that lncRNAs are now associated with, it is perhaps not surprising that there is growing evidence for their role in β-cell function and diabetes pathogenesis. Revealing their function will undoubtedly lead to a new wave of exciting targets to explore for therapeutic development.

## Conflict of Interest Statement

The authors declare that the research was conducted in the absence of any commercial or financial relationships that could be construed as a potential conflict of interest.
